# Amplify photo-avalanche nonlinearity beyond 500

**DOI:** 10.1038/s41377-025-01950-7

**Published:** 2025-08-19

**Authors:** Yawei Liu, Kai Liu, Hongjie Zhang

**Affiliations:** 1 State Key Laboratory of Rare Earth Resource Utilization, Changchun Institute of Applied Chemistry, Chinese Academy of Sciences, Changchun, China; 2 Department of Chemistry, Engineering Research Center of Advanced Rare Earth Materials (Ministry of Education), Tsinghua University, Beijing, China

**Keywords:** Optical materials and structures, Optical physics

*Nature* **643**, 669–674 (2025) 10.1038/s41586-025-09164-y

Photon avalanche is a distinctive optical nonlinear phenomenon observed in lanthanide-doped nanocrystals, which holds great potential for super-resolution imaging, ultrasensitive optical sensing, and multiphysics field detection. However, further enhancement of nonlinearity in photon avalanche nanomaterials remains challenging. Researchers from the National University of Singapore, Xiamen University, A*STAR in Singapore, and the National University of Singapore Suzhou Research Institute, have developed a very promising approach to amplifying the optical nonlinearity of photon-avalanche nanoparticles. They induced local crystal field distortions by modulating the internal sublattice structure inside the lanthanide-doped nanocrystals. This approach enhanced cross-relaxation between ions, while the engineered 27 nm nanocrystals enabled super-resolution imaging with a lateral resolution of 33 nm and an axial resolution of 80 nm using single-beam scanning microscopy, surpassing previous photon avalanche-based imaging techniques. Moreover, 176 nm nanodiscs exhibited an amplified optical nonlinearity exceeding 500, with this extreme nonlinearity effectively making the nanoparticles appear smaller than their physical dimensions during optical imaging, thus offering a pathway to overcome the traditional resolution limits imposed by probe dimensions.

